# Current Knowledge on Bee Innate Immunity Based on Genomics and Transcriptomics

**DOI:** 10.3390/ijms232214278

**Published:** 2022-11-18

**Authors:** Xiaomeng Zhao, Yanjie Liu

**Affiliations:** 1 College of Engineering, Hebei Normal University, Shijiazhuang 050024, China; 2 Key Laboratory for Insect-Pollinator Biology of the Ministry of Agriculture and Rural Affairs, Institute of Apicultural Research, Chinese Academy of Agricultural Sciences, Beijing 100093, China

**Keywords:** bees, innate immunity, pathogens, parasites, pesticides

## Abstract

As important pollinators, bees play a critical role in maintaining the balance of the ecosystem and improving the yield and quality of crops. However, in recent years, the bee population has significantly declined due to various pathogens and environmental stressors including viruses, bacteria, parasites, and increased pesticide application. The above threats trigger or suppress the innate immunity of bees, their only immune defense system, which is essential to maintaining individual health and that of the colony. In addition, bees can be divided into solitary and eusocial bees based on their life traits, and eusocial bees possess special social immunities, such as grooming behavior, which cooperate with innate immunity to maintain the health of the colony. The omics approach gives us an opportunity to recognize the distinctive innate immunity of bees. In this regard, we summarize innate bee immunity from a genomic and transcriptomic perspective. The genetic characteristics of innate immunity were revealed by the multiple genomes of bees with different kinds of sociality, including honeybees, bumblebees, wasps, leaf-cutter bees, and so on. Further substantial transcriptomic data of different tissues from diverse bees directly present the activation or suppression of immune genes under the infestation of pathogens or toxicity of pesticides.

## 1. Introduction

**Figure 1:**
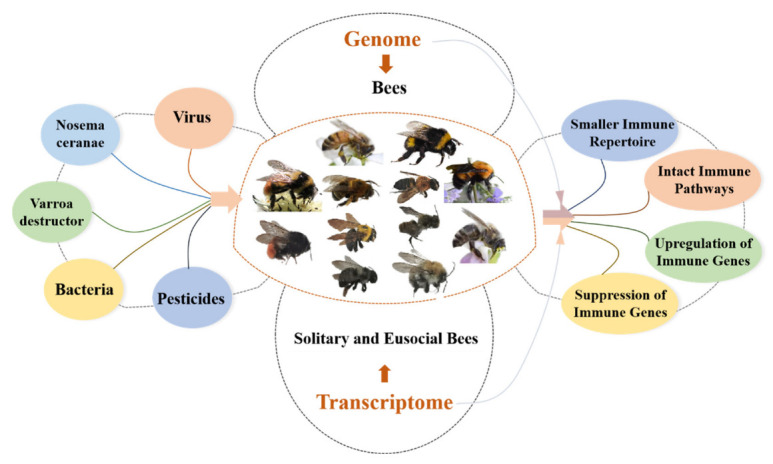
Overview of the innate immunity of bees from the genome and transcriptome perspectives.

While the demand for crop pollination by insects has tripled over the past 50 years, the pollinator-bee population has drastically declined due to climate change, habitat loss, emerging parasites and pathogens, and increased pesticide application [1,2,3,4,5,6]. Due to these challenges and environmental stresses such as poor nutrition and pesticide residues, pollinator bees use their innate immune system, which is their only defense, to maintain their individual health and that of the colony. The emergence and development of genomic and transcriptomic technology provide an opportunity to understand the mysteries of life sciences [7,8]. Similarly, genomic and transcriptomic research on bees have helped identify and understand the genetic traits of immunity and the immune response to environmental stressors and pathogens, both primordial aspects of the colony’s health [9,10]. This review focuses on the genetic characteristics of innate immunity and immune response to pathogens and pesticides in bees from a genomic and transcriptomic perspective (Figure 1). The present review outlines the uniqueness of innate bee immunity and immune genes in response to single or multiple threats to facilitate intensive study of the bee immune system.

## 2. Genomic Perspective of Innate Bee Immunity

The first complete bee genome, the *Apis mellifera* genome, was assembled and annotated in 2006 [11], and it was updated in 2014 and 2016 [12,13]. Compared to known genomes of model organisms such as *Drosophila melanogaster* and *Anopheles gambiae*, the *A. mellifera* genome encodes fewer immune proteins involved in the immune response process, starting from pathogen recognition to immune effectors. In fact, nearly two-thirds of the immune genes are reduced, but a small number of genes encode the components of the insect’s classical immune pathways, such as Toll, IMD, and JAK/STAT pathways [11,12,14]. Based on the genomic and transcriptomic analyses, *Apis cerana*, a species similar to *A. mellifera*, also possesses a small amount of innate immune genes and similar classical immune pathways compared to those of flies and mosquitoes, and most of its immune genes are similar to those of *A. mellifera* [15,16]. Recently, de novo genome assembly of Chinese plateau *A. cerana* has shown that the gene number of this genome is different from that of known *A. cerana* genomes [17]. As a representative of primary eusocial bees, bumblebee genomes from 17 species show that the major immune repertoire and immune gene number are both similar to those of *A. mellifera*, which is significantly lower compared with that of Dipteran models [18,19]. Moreover, another important Asian honeybee (*Apis dorsata*) genome also exhibits an immune repertoire similar to that of known bee genomes [20]. A reduced number of immune proteins might be seen as a result of the social immunity of social bees; eusocial and primary eusocial bees can cooperate to reduce disease transmission risk through their behavior, known as social immunity, which can be prophylactic or activated on demand [21]. However, expressed sequence tag (EST) databases of healthy and pathogen-challenged alfalfa leaf-cutting bee larvae have identified 104 putative immunity-related genes, including innate immune response genes that are highly conserved with honey bee genes, such as those involved in pathogen recognition, phagocytosis, prophenoloxidase cascade, melanization, coagulation, and several signaling pathways [22]. Similar smaller immune repertoires have been discovered in other available solitary or eusocial bee genomes, including those of *A. florea*, *Bombus terrestris*, *B. impatiens*, *Eufriesea Mexicana*, *Melipona quadrifasciata*, *Habropoda laboriosa*, *Megachile rotundata*, *Lasioglossum albipes*, and *Dufourea novaeangliae* [19,23]. Additionally, genomes of three parasitoid *Nasonia* species (*N. vitripennis*, *N. giraulti*, *and N. longicornis*) show an immune repertoire similar to that of *A. mellifera* but with a slightly higher gene count than that of the latter, although several immune genes are not yet identified [24]. Furthermore, a fig wasp (*Ceratosolen solmsi*) genome exhibits an immune repertoire and gene counts similar to those of *A. mellifera* [25]. Therefore, although different bee species possess slightly different immune gene counts, their innate immune system is characterized by integral immune pathways, and the reduced immune gene number is interestingly not related to the bees’ sociality [26]. Thus, genomic analysis is a powerful tool for exploring the innate immune components of both solitary and eusocial bees. Until now, several genomes of different bees have been determined, but the immune repertoire of these bees has to be further analyzed [27,28,29,30,31,32,33,34,35,36,37,38,39].

## 3. Transcriptomic Perspective of Innate Bee Immune Response

Transcriptomic analysis in bees indicates gene expression changes under certain conditions. For instance, the expression profile of the immune genes is mainly influenced by invasion by pathogens, such as viruses, bacteria, and parasites, as well as exposure to pesticides and other hazardous substances, as well as poor nutrition [40]. While nutrient status is key to an individual’s immune response, the relationship between nutrition and innate immunity is driven by energy consumption [41]. Pathogens adversely affect the health of wild and managed bees [42], and their infestation can trigger the innate immune response, thus blocking the infection and eliminating the pathogens [43]. In contrast, pesticides inhibit the innate immune response and promote pathogen spread and virulence, contributing to bee colony loss [3]. Hazardous substances such as nano- and micro-polystyrene plastics can disturb gut microbiota and inhibit intestinal immune response [44]. Regardless of the suppression or triggering of immune gene expression, transcriptomic analysis can directly reveal gene expression profile changes in different tissues of managed and wild bees infected by various pathogens and exposed to pesticides.

### 3.1. Immune Responses to Viruses

Bees can be infected by more than 20 viruses worldwide, most commonly by deformed wing virus (DWV), black queen cell virus (BQCV), Israeli acute paralysis virus (IAPV), and Sacbrood virus (SBV) [45,46,47]. Following an IAPV infection in *A. mellifera*, two immune genes involved in RNAi pathways Ago2 and Dicer, as well as other immune genes, were identified to be implicated in Toll and JAK/STAT pathways, and these findings overlap with those on immune gene response following other viral infections based on transcriptome analysis [48]. This analysis also demonstrated the dynamic changes in immune gene expression in the hours following an IAPV infection [49], and it has shown that BQCV infection triggers significant upregulation of immune genes such as those encoding antimicrobial peptides (abaecin, apidaecin, and hymenoptaecin), peptidoglycan recognition protein S2 (PGRP-S2), Ago2, and Dicer, (the latter two both implicated in RNAi pathways in *A. mellifera* brains [50]). A transcriptomic analysis of larvae and pupae has revealed changes in immune genes involved in antimicrobial peptides (AMPs) and melanization pathways following DWV and SBV infection in *A. mellifera*; both are positive-strand RNA viruses and members of the iflavirus group [51]. In SBV-carrying *A. mellifera* larvae, approximately 20 differentially expressed immune-related genes have been identified [52]. In *A. cerana* larvae naturally infected with CSBV, small interfering RNA-targeting serine proteases that are involved in the immune response are upregulated [53]. Moreover, transcriptomic analysis has revealed that the sirtuin signaling pathway may be a novel mechanism of immune response to CSBV infection in honeybees [54] and that the immune genes for AMPs, Ago2, and Dicer are involved in the innate immune response to DWV infection in *A. mellifera* brains [55]. The transcriptome profile of *A. mellifera* eggs shows the trans-generational effects of SBV and DWV on several gene expression levels, indicating the different virulence of DWV and SBV during vertical transmission [56]. Bee viruses can be transmitted by *Varroa destructor* mites, which drives changes in virus distribution, prevalence, and virulence [57]. Transcriptomic analysis shows that *varroa*-induced viral replication is closely related to the expression of immune genes *PGRP-S2*, *NimC2*, and *Eater-like* as well as serine protease levels in *A. mellifera* adults [58]. Furthermore, transcriptomic analysis revealed that the Varroa mite alone and the DWV coupled with the mite could induce upregulation of different immune genes involved in the Toll and JNK pathways, respectively [59]. In addition, multiple transcriptome data have shown that *hymenoptaecin*, *defensin-2*, *PGRP-S1*, and *B-gluc1* are common host immune genes that respond to the major pathogens and parasites such as RNA viruses, *V. destructor*, *N. apis*, and *N. ceranae* in *A. mellifera* [60]. Meanwhile, despite the fact that some common genes are identified above, important differences in the transcription responses of honey bees to various pathogens were revealed [60].

### 3.2. Immune Response to Parasites

Along with acting as a virus vector, the parasitic *Varroa destructor* also reduces nutrient levels and suppresses individual immune function, and is an underestimated parasite threatening the health of bee colonies [41,61]. Transcriptomic analysis has shown that immune gene expression levels change as a response to the mite *V. destructor* (e.g., *PGRP-S3*, *GNBP1*, Toll receptors, and serine protease) [62]. Updated transcriptomic analysis of newly emerged *A. mellifera* has identified three immune genes encoding PGRP-2, hymenoptaecin, and glucan recognition protein, which could be good candidates as markers for immune response to *Varroa* infestation [63]. Moreover, *Varroa* parasitism could also cause downregulation of autophagic-specific gene 18 and poly (U) binding factor 68 Kd (pUf68), and Rab7 upregulation in *A. mellifera* [64]. A set of genes related to social immunity has been identified in *A. mellifera* by analyzing the comparative transcriptome of *varroa*-hygienic bees [65]. Nutrigenomics shows that pollen and sugar supplements positively affect the production of some AMPs but cannot reverse the harmful effects of *varroa* parasitism [66].

Additionally, based on the transcriptomic data, the expression of immune genes encoding serine protease, lysozyme 1, and hymenoptaecin is found to be suppressed by *Nosema ceranae* infection in *A. mellifera* [67]. Serine proteases, peptidoglycan recognition proteins, and antimicrobial peptides are downregulated following *N. ceranae* infection in *A. mellifera* [68]. Besides the differently expressed immune genes, the whole transcriptome has also identified the *N. ceranae* infection-related long non-coding RNAs (lncRNAs) that may participate in the *A. mellifera* immune response [69]. Comparative transcriptome analysis has identified the genes involved in cellular immune pathways, such as ubiquitin-mediated proteolysis, endocytosis, lysosomes, phagosomes, autophagy, and melanogenesis, and in humoral immune pathways, such as MAPK, JAK/STAT, and Toll/IMD signaling pathways, in *N. ceranae-*infected *A. cerana* [70]. Moreover, transcriptome analysis has identified CircRNAs targeting mRNAs that were annotated to cellular immunity pathways, including endocytosis, lysosomes, and phagosomes in the gut of *N. ceranae*-infested *A. cerana* [71]. Except for honeybee, many genes, including those encoding receptors (GNBPs), signaling pathway components, and AMPs, have been identified in *Bombus terrestris* infested by *Crithidia bombi*, and these genes are closely related to canonical immune pathways [72]. Transcriptomic analysis of *Sphaerularia bombi-*infected *B. terrestris* queens during and after diapause showed that increased expression of immune genes (e.g., genes encoding scavenger receptors, Toll-like receptors, domeless, C-type lectin, and draper) is mainly induced by *S. bombi* after diapause [73]. Interestingly, the transcriptome has been used to evaluate the role of pathogens and pesticides in reducing the *B. terricola* population by detecting immune and detoxification genes [74].

### 3.3. Immune Response to Bacteria

Genome microarrays demonstrate that immunostimulants such as bacterial infection and wounds could induce hundreds of significantly differentially expressed genes, including the previously identified canonical immune genes and other major unidentified new genes [75]. Transcriptomic analysis showed that the expression levels of *hymenoptaecin*, *apidaecin*, and *defensin-1* are significantly upregulated in *A. mellifera* larvae infested with the bacterial pathogen *Paenibacillus* [76]. Transcriptome profiling has revealed an upregulation of immune-related genes, such as those encoding Toll-like receptors, integrin, and antimicrobial peptides, in *Ascosphaera apis*-infected *A. mellifera* larvae [77]. Moreover, 13 differently expressed immune genes involved in humoral and cellular immunity were identified in the *A. mellifera* gut following *A. apis* infestation [78]. Transcriptomic analysis of the *A. cerana* larval gut showed upregulation of immune genes such as humoral and cellular immune genes following *A. apis* infestation [79]. In addition to the pathogenic bacteria, the beneficial gut microbe *Frischella perrara* can strongly activate the host immune response and upregulate important immune genes, including those encoding pattern-recognition receptors, antimicrobial peptides, transporter genes, and melanization cascade in *A. mellifera* [80]. Interestingly, transcriptome analysis has shown that the gut microbe *Lactobacillus apis* triggers the expression of *PGRP-S3*, *Spätzle*, and antibacterial proteins, which can inhibit infection by *Hafnia alvei*; further genomic analysis suggested that the S-layer proteins of *L. apis* are potentially involved in honeybee Toll signaling and in the activation of antibacterial protein production in honeybees [81]. The gut microbiota can be altered by polystyrene microplastic exposure and might influence the expression of gut immune genes; for instance, it can cause an upregulation of *apidaecin* and *abaecin* and dose-dependent downregulation of *domeless*, *hopscotch*, and *symplekin* in *A. mellifera* [82]. Another *A. mellifera* gut transcriptome has shown that microplastic polystyrene ingestion triggers upregulation of PGRP-S3, defensin-2, and dose-dependent differently expressed genes encoding Toll-like receptors, PGRP-S2, defensin-1, hymenoptaecin, and apidemins [83]. Additionally, as a parasitoid wasp differing from honeybees [84], transcriptomic analysis suggests that *Nasonia vitripennis* may possess novel immune components against bacterial infection [85]. As an important model system, the transcriptome of *N. vitripennis* will contribute to our comprehensive understanding of innate bee immunity.

### 3.4. Immune Suppression Due to Pesticides

Based on a transcriptomic analysis, five differently expressed immune genes encoding hymenoptaecin, abaecin, apidaecin, apisimin, and lysozyme are found in *A. mellifera* larvae exposed to sublethal levels of imidacloprid [86]. Another transcriptome analysis has identified immune-related genes (*abaecin*, *eater*, *hymenoptaecin*, *defensin1*, *defensin2*, *vitellogenin*, and *apidaecin*) involved in the immune response against neonicotinoids such as imidacloprid and clothianidin in honeybees; it has also shown that *abaecin* and *hymenoptaecin* expression levels are significantly higher in neonicotinoid-exposed *A. cerana* than in neonicotinoid-exposed *A. mellifera* [87]. Moreover, a transcriptomic analysis also demonstrated that imidacloprid could alter the innate immune gene expression of brain tissue in the bumblebee *B. terrestris* [88]. Meanwhile, following exposure to sublethal doses of imidacloprid and deltamethrin, detoxification genes are upregulated, and immune genes encoding apidaecin and hymenoptaecin are significantly downregulated in the brain tissue of *A. mellifera* [89]. Another transcriptomic analysis has indicated that environmentally relevant concentrations of the neonicotinoid clothianidin can induce the downregulation of scavenger receptor class B member 1 and upregulation of hymenoptaecin and apidaecin, while imidacloprid can cause hymenoptaecin upregulation in the brain tissue of *A. mellifera* [90]. Chronic oral exposure to the neonicotinoid clothianidin may alter the expression of immune defense-related genes by upregulating exosome complex component RRP46 and downregulating C-Maf-inducing protein-like in worker bees but not in male *B. impatiens* [91]. Moreover, immune gene expression following *V. destructor* mite infestation differs from that following exposure to the neonicotinoid insecticide clothianidin in a single *A. mellifera* colony [92].

Transcriptome analysis has also helped identify the impacts of various pesticides on bee immunity. For instance, immune genes encoding defensin1, vitellogenin, and scavenger receptor class B member 1 are shown to be downregulated in thiamethoxam-treated *A. mellifera* brain tissues [90,93], while innate immunity-related proteins like apolipophorin-III-like proteins are significantly upregulated in the brains of *A. mellifera* exposed to environmental concentrations of the neonicotinoid thiacloprid [94]. Additionally, dinotefuran treatment significantly affects the expression of immune-related genes such as those encoding glutathione S-transferase S4, prolactin-releasing peptide receptor, defensin 2 (Def2), and vesicle-associated membrane protein 2 in the brains of *A. mellifera* [95]. The immune genes and expression levels of that in response to four insecticides, namely chlorpyrifos, malathion, cypermethrin, and chlorantraniliprole, are different but the differently expressed immune genes are all involved in the IMD pathway and production of AMPs in *A. mellifera* [96]. Transcriptome analysis of midguts also identified differentially expressed genes involved in immunity in nitenpyram-treated *A. mellifera* [97]. Dimethoate, an insecticide, can cause apisimin and Toll downregulation, and flupyradifurone or chlorantraniliprole may induce defensin1 and processing enzyme downregulation in the larvae of *A. mellifera* [98]. Differently expressed immune genes are identified in *A. mellifera* under benomyl stress [99]. Transcriptomic analysis has revealed that several immune genes such as those encoding abaecin, Def1, SP28, Toll-1, Toll-6, Toll-8, Toll-10, and MyD88 are upregulated in benomyl-treated *A. mellifera* [100]. Additionally, pesticides (acaricides) used to treat varroa mites in bee colonies can also induce *Dscam* downregulation, an immune gene important to cellular immunity, and *basket* downregulation, an orthologue of JNK signaling, in *A. mellifera* [101]. Interestingly, transcriptomic analysis of different tissues suggests that AMPs (e.g., apisimin and defensin) are simultaneously expressed with nectar processing enzymes in the hypopharyngeal and mandibular glands of foragers but not in the nurses of *A. mellifera* as a response to potential environmental threats during nectar and pollen collection [102].

## 4. Conclusions

Advances in genomic and transcriptomic analyses permit recognizing the fundamental genetic characteristics of bees and help understanding of gene expression changes as part of the response to various pathogens and/or internal or external environmental stressors. Multiple bee genomes have revealed that a small number of immune genes are involved in classical insect immune pathways in the bee’s innate immunity system. Based on genomes, the transcriptomic analysis has revealed some of the immune genes acting as a response to various pathogens such as viruses, bacteria, and parasites; these genes are suppressed by hazardous pesticides. However, more in-depth studies are needed to identify more immune genes critical to the immune response against threatening factors and maintain the bee colony’s health. Indeed, recognizing these immune genes provides a basis for the subsequent elaboration of the function and structure of these genes by other molecular biological methods. Moreover, further research is needed for a more comprehensive understanding of innate bee immunity. Future genomic analysis of different species and transcriptomic analysis of different tissues following various internal and/or external environment stressors could help identify related immune genes.

## Data Availability

Not applicable.
